# Myelin oligodendrocyte glycoprotein (MOG) associated optic neuritis in a patient with idiopathic intracranial hypertension (IIH) and compressive optic neuropathy case report

**DOI:** 10.1186/s12886-023-03280-x

**Published:** 2024-02-13

**Authors:** Ryan D. Sorensen, Ashtyn Vogt, Noor Laylani, Mohammad Pakravan, Andrew G. Lee

**Affiliations:** 1 Baylor College of Medicine, 2535 Shakespeare St Unit 2, 77030 Houston, TX USA; 2 Texas A and M College of Medicine, Dallas, TX USA; 3 Department of Ophthalmology, Blanton Eye Institute, Houston Methodist Hospital, Houston, TX USA; 4 Departments of Ophthalmology, Neurology, and Neurosurgery, Weill Cornell Medicine, New York, USA; 5 Department of Ophthalmology, University of Texas Medical Branch, Galveston, TX USA; 6 University of Texas MD Anderson Cancer Center, Houston, TX USA; 7 Texas A and M College of Medicine, Bryan, TX USA; 8 Department of Ophthalmology, The University of Iowa Hospitals and Clinics, Iowa City, IA USA

**Keywords:** Idiopathic intracranial hypertension, Myelin oligodendrocyte glycoprotein antibody, Pituitary adenoma

## Abstract

**Background:**

Myelin oligodendrocyte glycoprotein-associated disease (MOGAD) has a wide phenotypic expression and should be considered in a differential diagnosis of patients with optic disc edema and increased intracranial pressure because MOGAD can mimic IIH and compressive optic neuropathy.

**Case presentation:**

A 53-year-old woman with a history of presumed idiopathic intracranial hypertension (“IIH”) presented with new headache and visual loss. She had a BMI of 35.44 kg/m2 and a past medical history significant for depression, hepatitis C, hyperlipidemia, and uterine cancer post-hysterectomy. She had undergone multiple lumboperitoneal shunts for presumed IIH and had a prior pituitary adenoma resection. Her visual acuity was no light perception OD and counting fingers OS. After neuro-ophthalmic consultation, a repeat cranial MRI showed symmetric thin peripheral optic nerve sheath enhancement of the intra-orbital optic nerves OU. Serum MOG antibody was positive at 1:100 and she was treated with intravenous steroids followed by plasma exchange and rituximab.

**Conclusions:**

This case highlights the importance of considering MOGAD in the differential diagnosis of optic neuropathy. Although likely multifactorial, we believe that the lack of improvement in our case from presumed IIH and despite adequate neurosurgical decompression of a pituitary adenoma with compression of the optic apparatus reflected underlying unrecognized MOGAD. Clinicians should consider repeat imaging of the orbit (in addition to the head) in cases of atypical IIH or compressive optic neuropathy especially when the clinical course or response to therapy is poor or progressive.

## Background

Myelin oligodendrocyte glycoprotein-associated disease (MOGAD) has a wide phenotypic expression that includes optic neuritis, transverse myelitis, acute demyelinating encephalomyelitis (ADEM), as well as other neurologic syndromes. MOG antibody (MOG-Ab) is an autoantibody that has been associated with neuromyelitis optica spectrum disorder (NMOSD), optic neuritis (ON), and serous retinal detachment [1–4]. We report a case of MOGAD optic neuritis in a patient with presumed idiopathic intracranial hypertension (IIH) and compressive optic neuropathy who did not recover vision despite aggressive surgical treatment for both IIH and compressive optic neuropathy. We hypothesize that MOGAD mimicked these conditions in our case.

## Case presentation

A 53-year-old woman presented with bilateral visual loss. She had a prior history of “IIH” by modified Dandy criteria 19 years prior but aggressive surgical treatment with lumboperitoneal shunt placement and two revisions failed to improve the vision. She had a pituitary adenoma that was resected but the vision did not improve and she presented with 3 weeks later with new-onset headache and visual loss. Her BMI was 35.44 kg/m^2^. Past medical history included depression, hepatitis C, hyperlipidemia, and uterine cancer post-hysterectomy. The patient was taking trazodone for anxiety and buprenorphine for chronic pain. She had allergies to penicillin, tolterodine, ondansetron, metoclopramide, and prochlorperazine. She never smoked and had no history of alcohol or drug use. The family history was non-contributory.

At presentation she reported new headaches, transient visual obscurations, and a history of “papilledema” and “IIH” in 2003, at the age of 35. In 2003, cranial magnetic resonance imaging (MRI) was normal and lumbar puncture (LP) revealed normal CSF contents but an elevated opening pressure, which was compatible with the modified Dandy criteria for IIH. The patient had progressive visual loss that required LP shunt and had a subsequent shunt revision.

From 2015 to 2018, she began experiencing painless peripheral vision loss, which she and her physicians attributed to “old damage from the pseudotumor cerebri”.

**Fig. 1:**
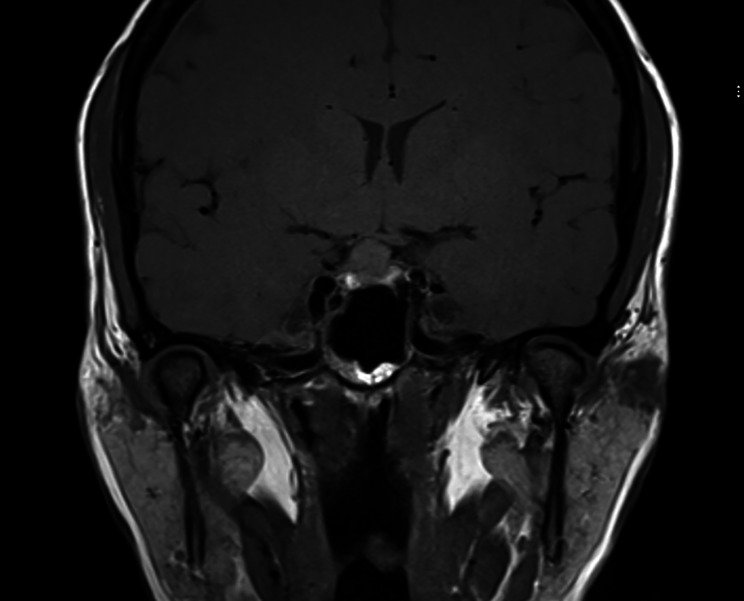
Coronal T1 preoperation. Small possibly necrotic slow-enhancing component in the inferior aspect of the pituitary gland

The patient presented again in 2018 with progressive peripheral vision loss in both eyes. A repeat cranial MRI showed a prior stable mild Chiari I malformation but a new suprasellar pituitary adenoma (Fig. 1). A diagnosis of compression optic neuropathy was made. Visual acuity was hand motion in the right eye (OD) and 20/60 in the left eye (OS). Automated perimetry (Humphrey visual field (HVF) 24 − 2) revealed a temporal hemianopic impairment with a superimposed inferior altitudinal field loss OS and diffuse depression OD. Repeat LP results revealed normal CSF and opening pressure. The patient had a Grade 1 pituitary adenoma removed completely via a transnasal technique, but her vision did not improve, likely due to the prior optic atrophy that was present for years prior to resection.

**Fig. 2:**
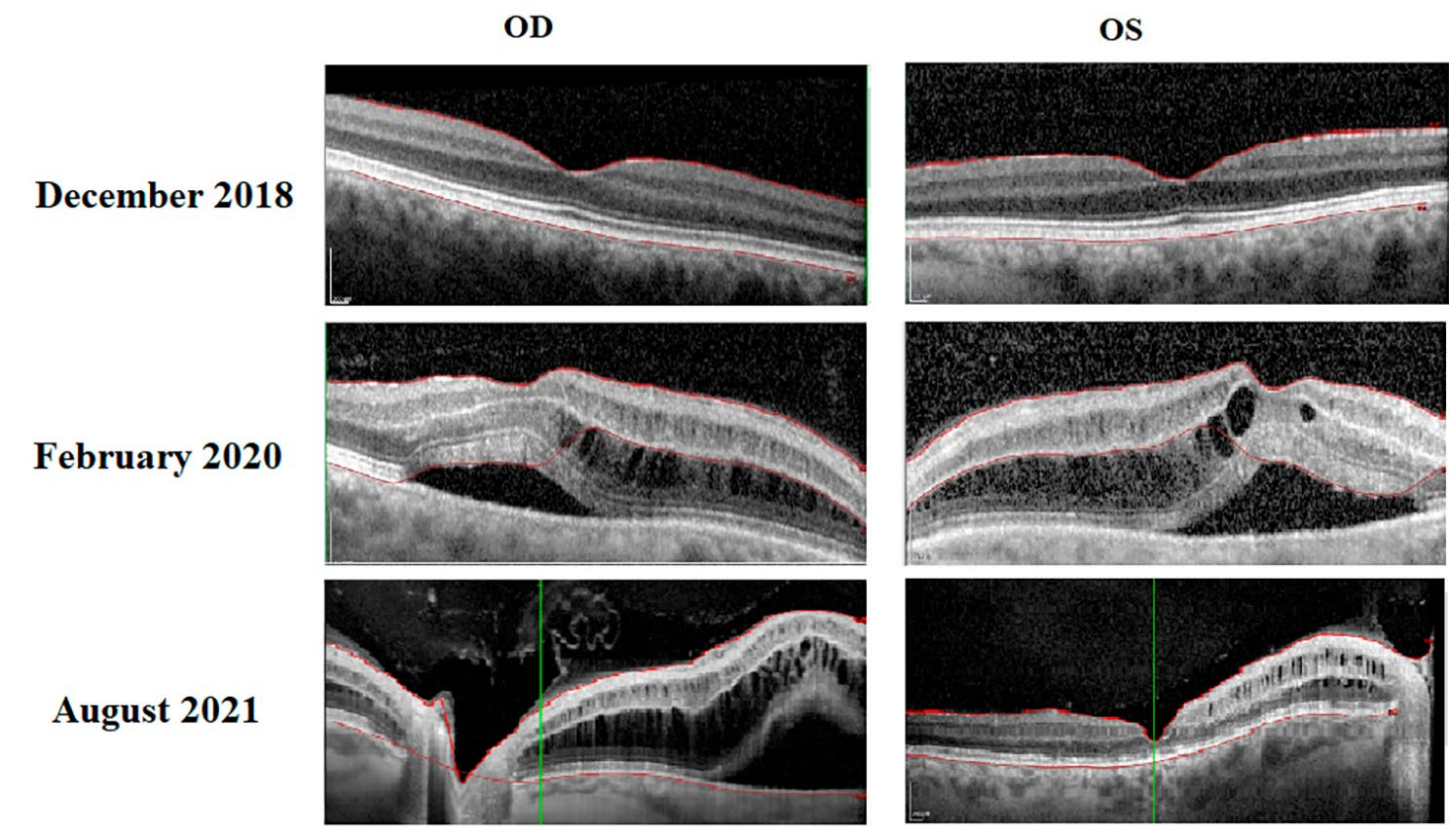
OCT scans from 2018–2021

In February 2020 the patient presented to the neuro-ophthalmology clinic at Houston Methodist Hospital with one-month worsening headaches and vision loss for which she was admitted. On neuro-ophthalmic examination, the visual acuity was no light perception OD and counting fingers OS. The right pupil was amaurotic. Optical coherence tomography (OCT) of the retinal nerve fiber layer and the macular ganglion cell layer showed diffuse loss OU. New subretinal and intraretinal edema was seen on OCT (Fig. 2) and ophthalmoscopy showed diffuse optic atrophy OU.

**Fig. 3:**
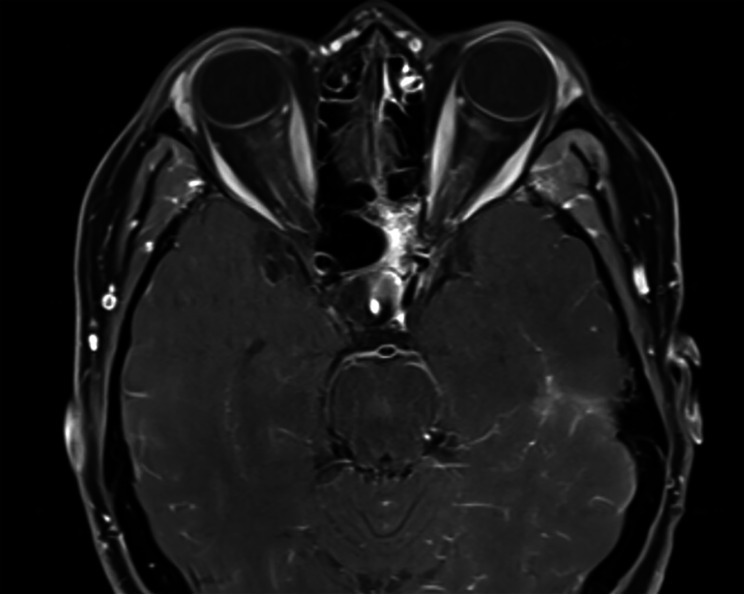
Axial T1 fat-suppressed magnetic resonance imaging shows optic nerve sheath enhancement bilaterally

Repeat cranial MRI showed gross total resection of the pituitary adenoma with no recurrent or residual disease. LP showed normal opening pressure and CSF content. Repeat orbital MRI with contrast however showed symmetric thin peripheral optic nerve sheath enhancement of the intra-orbital optic nerves OU (Fig. 3). Serum MOG-Ab testing was positive at a 1:100 titer. She was treated with intravenous steroids followed by plasma exchange and rituximab. There was an improvement of subretinal fluid collection following the plasma exchange, and she continued rituximab every 6 months. In September of 2020 her VA was NLP OD and 20/400 OS but the incomplete improvement was attributed to prior optic atrophy. In August of 2021 OCT was done (Fig. 2) and her VA was stable at NLP OD and 20/400 OS. On May 3, 2022, she presented with 3 weeks of worsening vision and headaches, her VA was NLP OD and CF OS, with a right RAPD, on OCT global was 75 OD and 67 OS after being 99 OD and 84 OS and showed bilateral optic atrophy despite treatment for MOGAD likely due to the delayed treatment of MOGAD.

## Discussion and conclusions

Both IIH and MOGAD-related optic neuritis can present with visual loss, headache, and optic disc and macular edema. There have been previous cases of MOGAD patients with typical symptoms who were initially diagnosed with IIH but then developed visual deficits suggestive of optic neuritis, in certain clinical contexts (e.g., fulminant IIH) it may be difficult to initially differentiate visual loss from papilledema due to IIH and MOGAD optic neuritis [1, 2]. Table 1 lists the prior cases of MOGAD associated with increased intracranial pressure mimicking IIH. The proposed mechanism for MOGAD-related increased intracranial pressure has been CSF inflammation and secondary decreased CSF reabsorption [2]. We believe that this patient had MOGAD with secondary increased intracranial pressure in the setting of a concomitant pituitary adenoma. Although the optic neuropathy is likely multifactorial, we hypothesize that MOGAD was the primary cause for the visual loss and that the presentation mimicked the visual loss from prior “IIH” and then later from pituitary adenoma.

**Table 1:** Prior cases of MOGAD associated with increased intracranial pressure mimicking IIH compared to the case presented

	PresentingCase	Patient(1) from“Case of.atypical Anti-MOG Syndrome” [1]	Patient(2) from “Case of atypical Anti-MOG Syndrome” [1]	“Atypical MOG Presenting with isolated elevate ICP” [2]	“Caseof MOG associated with serious retinal detachment” [3]	Patient(1) from “MOG masquerading as IIH” [4]	Patient(2) from “MOG masquerading as IIH” [4]	Patient(3) from “MOG masquerading as IIH” [4]	Patient(4) from “MOG masquerading as IIH” [4]	Patient(5) from “MOG masquerading as IIH” [4]	CaseAverages from MOG Clinical Characteristics [5]
**Gender**	Female.	Female.	Male.	Male.	Female.	Female.	Male.	Female.	Male.	Female.	57% Female.
**BMI**	35.44 kg/m2	Obese	NA	Obese	NA	38 kg/m^2^	32 kg/m^2^	41 kg/m^2^	32 kg/m^2^	23 kg/m^2^	NA
**Visual Acuity**	HA OD and 20/60 in OS.	20/400 OD CF 2 OS.	HA OD. RAPD OD.	Normal.	20/50 OD 20/40 OS.	Mildly decreased.	Mildly decreased.	20/60 bilaterally.	Unable to obtain.	20/60 OD. RAPD OD.	20/30 Bilateral.
**Visual Fields**	Diffusely depressed OD and a temporal hemianopia superimposed on inferior altitudinal field defect OS.	Inferior and supero-temporal visual field defects OS.	NA	Normal.	Mild enlargement of the physiological blind spot OS.	Bilaterally enlarged blind spots.	Bilaterally enlarged blind spots.	Nasal step defect in one eye and a normal field in the other.	NA	NA	NA
**OCT**	OCT 37 OD 40 OS with diffuse optic atrophy. Edema in macula.	NA	NA	NA	OCT revealed subretinal fluid in macula bilaterally.	Thickened peripapillary retinal nerve fiber layer (RNFL) bilaterally.	Thickened RNFL bilaterally.	Thickened RNFL bilaterally (RE 140 μm, LE 172 μm).	Thickened RNFL bilaterally.	NA	NA
**Fundoscopic Findings**	Intraretinal cystic change in the inner retina corresponding with optic atrophy.	Relative red desaturation OS. Bilateral papilledema.	Disk edema OD.	Bilateral papilledema.	Bilateral disk edema.	Bilateral disc edema.	Bilateral disc edema.	Bilateral disc edema.	Bilateral disc edema.	Bilateral disc edema.	36/42. Present in 86% of cases.
**Symptoms**	Painless progressive peripheral vision loss.	Bifrontal headaches, photophobia, blurry vision.	Headache, neck pain, neck stiffness, photophobia, nausea, vomiting.	Headache, blurry vision, nausea, and vomiting.	1-week history of blurred vision.	Headaches, blurred vision.	Headaches, blurred vision.	Headaches, blurred vision.	Headaches, blurred vision.	Headaches, blurred vision.	Pain (86%) Recurrent optic neuritis (10%) Monophasic optic neuritis (10%) ADEM (18%) NMSOD-like phenotype (22%)
**MRI findings**	ONSE.	Bilateral swelling and increased FLAIR with enhancement of the optic nerves. An enhancing pituitary mass.	Inflammation of the right optic nerve. Non-specific bilateral periventricular white matter changes.	Signs of elevated ICP, dilation of optic nerve sheaths, flattening of posterior globes, and a partially empty sella.	ONSE.	Hyperintense signal in both optic nerves with gadolinium enhancement of the right optic nerve.	ONSE.	ONSE.	ONSE.	Enhancement of the right optic nerve. Several hyperintense periventricular and subcortical white matter lesions.	Perineural enhancement: 50% Length involved more than half: 80% Orbital portion involved: 92% Intracranial portion involved: 72% Chiasm involved: 12%
**CSF Opening Pressure and Analysis**	Markedly elevated. Normal constituents	29 cmH_2_O Lymphocytic Pleocytosis	24 cmH_2_O Polymorphonuclear Pleocytosis	52 cmH_2_O Lymphocytic Pleocytosis	NA	26 cmH_2_O Normal constituents	33 cmH_2_O Normal constituents	23 cmH_2_O Normal constituents	24 cmH_2_O Normal constituents	35 cmH_2_O Normal	44% had pleocytosis
**MOG Titer**	1:100	1:100	Positive.	1:10,000	1:512	Positive.	Positive.	Positive.	Positive.	Positive.	100*

NA - not available, HA - Hand motion, CF - count fingers, ONSE - Optic Nerve Sheath Enhancement, *Criteria for study was MOG positive titer.

MOGAD can present bilaterally and with optic disc edema, in contrast to typical optic neuritis from multiple sclerosis which is usually unilateral and associated with a normal fundus or mild if any optic disc edema. In addition, optic nerve sheath enhancement has been shown to be a differentiating and distinctive finding for MOGAD (see table) that is not typically seen in IIH or MS-related optic neuritis [5]. Finally, although macular edema can rarely be seen in optic neuritis this finding has been reported in MOGAD.

This patient has a very unusual case of MOG-related optic neuritis which was complicated by the presence of a pituitary adenoma 15 years after the initial presenting symptoms. Either MOG-related optic neuritis manifested as IIH from the beginning of this patient’s history or it developed in the setting of a long-standing IIH. Clinicians should be aware that IIH symptoms (e.g., fulminant IIH) can mirror MOGAD-related optic disc edema. Orbital and cranial MRI should be performed to evaluate for optic nerve sheath enhancement which can be more suggestive of MOGAD than IIH. In addition to having a positive titer, LP may also exhibit inflammatory CSF indices, which could be the cause of the elevated ICP caused by MOGAD, however, there are many cases reported of MOGAD masquerading as IIH which had no inflammatory markers noted in the CSF as shown in Table 1 similar to this patient’s first CSF study [4, 5].

We believe that our case highlights the importance of considering MOGAD in the differential diagnosis of any acute bilateral optic neuropathy. Acute fulminant presentations of IIH can produce a similar clinical picture to MOGAD. Likewise lack of visual improvement despite adequate neurosurgical decompression of a pituitary adenoma should also prompt consideration for testing for MOGAD. In addition, clinicians should consider imaging of the orbit and brain with contrast in cases of fulminant, progressive, or atypical IIH and in cases of compressive optic neuropathy which do not recover following surgical decompression. The presence of optic nerve sheath enhancement on orbital MRI should prompt consideration for MOGAD.

## Data Availability

The images in the figures are from the patient’s medical record and are used with the written consent of the patient. Additional data is available upon request.

## Abbreviations

MOGAD: Myelin oligodendrocyte glycoprotein-associated disease
ADEM: Acute demyelinating encephalomyelitis
MOG-Ab: Myelin oligodendrocyte glycoprotein antibody
NMOSD: Neuromyelitis optica spectrum disorder
ON: Optic Neuritis
IIH: Idiopathic intracranial hypertension
